# Retraction: How the Malaria Vector *Anopheles gambiae* Adapts to the Use of Insecticide-Treated Nets by African Populations

**DOI:** 10.1371/journal.pone.0156196

**Published:** 2016-05-19

**Authors:** Mamadou Ousmane Ndiath, Catherine Mazenot, Cheikh Sokhna, Jean-François Trape

The authors of this article have requested retraction on the basis of concerns about the experimental work and the integrity of the reported data.

An institutional investigation was conducted; the enquiry did not locate the samples which underlie the analyses reported in the article and was unable to verify that the mosquito dissections had taken place. In light of the findings from the enquiry, the direction of the Institut de Recherche pour le Développement support the retraction of the article.

In light of the above concerns, the authors retract this publication.
